# Atmospheric Pollution and Hospitalization for Cardiovascular and Respiratory Diseases in the City of Manaus from 2008 to 2012

**DOI:** 10.1155/2020/8458359

**Published:** 2020-04-01

**Authors:** Daniel S. Sacramento, Lourdes C. Martins, Marcos A. Arbex, Ysabely de A. P. Pamplona

**Affiliations:** ^1^Manaus Municipal Health Secretariat, Manaus 69057-001, Brazil; ^2^Department of Public Health, Catholic University of Santos, Santos 11015-008, Brazil; ^3^Department of Medicine, University of Araraquara, Araraquara 14801-340, Brazil

## Abstract

**Objective:**

To relate the levels of air pollution and hospital admissions for cardiovascular and respiratory diseases in the city of Manaus in Brazil from 2008 to 2012.

**Method:**

This is an ecological time-series study among children (under 5 years of age) and elderly (above 60 years of age). Data on the daily number of hospitalizations for cardiovascular and respiratory diseases, pollutants (PM_2.5_), temperature, and humidity were used. Poisson generalized additive models were used to estimate the association between variables. Increases in hospitalizations for cardiovascular and respiratory diseases were estimated for the interquartile range (IQR) daily mean level of each variable studied, with a confidence interval of 95%.

**Results:**

Respiratory diseases and children: −0.40% (95% CI: −1.11, 0.30), 0.59% (95% CI: −0.35, 1.52), and 0.47% (95% CI: −3.28, 4.21) for PM_2.5_, temperature, and humidity, respectively. Respiratory diseases and elderly: 0.19% (95% CI: −0.93, 1.31), −0.10% (95% CI: −1.85, 1.65), and −6.17% (95% CI: −13.08, 0.74) for PM_2.5_, temperature, and humidity, respectively. Cardiovascular diseases and elderly: −0.18% (95% CI: −0.86, 0.50), −0.04% (95% CI: −1.10, 1.03), and −3.37% (95% CI: −7.59, 0.85) for PM_2.5_, temperature, and humidity, respectively.

**Conclusions:**

The time-series study found no significant association between PM_2.5_, temperature, humidity, and hospitalization, unlike the evidences provided by the present academic literature. Since there is no air quality monitoring network in Manaus and the option available in the present study was to reproduce some information obtained from remote sensing, there is a need for implementation of ground monitoring stations for health and environmental studies in the region.

## 1. Introduction

The interaction between men and the surrounding environment has promoted the exposure to several aggressors, either natural or resulting from anthropogenic activity. Although the effects of pollution have been reported since antiquity, pollution affects population in large proportions solely from the beginning of the industrial revolution [1]. Exposure to air pollutants may influence humans' health since intrauterine life. Studies have shown that pollutants have deleterious effects even at exposure levels below the acceptable standard limits. The most affected and susceptible age groups are children and the elderly [2–4].

In the Brazilian Amazon, urbanization has been a rapid process. The Brazilian Amazon covers the states of Acre, Amapá, Amazonas, Pará, Rondônia, Roraima, Tocantins, Mato Grosso, and part of Maranhão and hosts 12.8% of the national population. The fast process of urbanization coupled with significant changes in the pattern of exploitation of natural resources has caused many types of environmental impacts, including loss of biodiversity, reduction of the productive potential of soils, erosion, river pollution, deforestation, and forest fires. These impacts have altered the relationship between urban and rural spaces, resulting in the emergence of new territorialities with positive and/or negative configurations [5]. Urban air pollution produced mainly by forest fires and increased vehicular fleet, as well as the installation of industries in the region (especially the Industrial Pole of Manaus), constitutes one of the negative configurations that these new territorialities can assume.

Currently, air quality monitoring is an object of research in several developed or developing countries. In the Amazon, environmental information databases in several areas are affected by the lack of spatial and environmental coverage. In turn, the lack of data for continuous air quality monitoring and forecasting can translate into consequences for public health [6]. Studies on the effects of air pollution in the Amazon have mostly focused on the analysis of the effects of biomass burning on outpatient care and the number of hospitalizations for respiratory diseases, mainly asthma [7].

As exposure to pollution and weather conditions occur simultaneously, it is important to seek to understand not only their isolated effect but also their interaction, investigating whether they modify the effect of each other. The combination of risks may follow simple or complex patterns and may vary among different geographic regions. The characterization of such a combination of factors may clarify many questions through the description of more realistic estimates of risk and the establishment of new guidelines in public health.

Estimating the health risks caused by air pollution in the population is a first step for the planning and implementation of actions aimed at a healthier environment. The production of this type of knowledge is fundamental for the formulation of public policies that promote socioeconomic development and that consider environmental issues and people's quality of life. Thus, the objective of this study was to relate levels of air pollution to hospital admissions for cardiovascular and respiratory diseases in the city of Manaus from 2008 to 2012.

The present study is structured into five sections besides the introductory one.  Section 2 presents the literature review about air pollution and health risks.  Section 3 presents the materials and methods.  Section 4 presents the results.  Section 5 presents the discussion. Finally, the conclusions are presented.

## 2. Literature Review

Air pollution is recognized as a major risk factor for population health [8]. Exposure to air pollution represents the largest and greatest health risk from the environment and is among the nine major modifiable risk factors, ahead of other common factors such as physical inactivity, high cholesterol, and drug use. Its presence is still associated with a significant reduction in life expectancy and productivity [9].

Particulate matter (PM) is the most common pollutant associated with adverse health effects [10]. PM is composed of carbonaceous particles associated with reactive and organic metals, including nitrates, sulphates, polycyclic aromatic hydrocarbons, endotoxins, and metals such as iron, copper, zinc, nickel, and vanadium [11]. Its composition varies depending on the geographical location in each country, the type of industry existing, and the amount of fossil fuel burned for heating and transport [12].

The concentration of particulate matter present in pollution influences the disease and where it will accumulate in the human body upon inhalation. PM_2.5_ lodges in the lungs and smallest airways. PM_10_ tends to deposit in the upper respiratory tract and upper airways. Particle size also influences the adverse effects of its exposure. Smaller particle sizes have a greater impact on health due to their ability to reach smaller airways; epidemiological studies have focused on the effects of PM_2.5_ and PM_10_ [13]. PM_2.5_ has different health effects than PM_10_ because of its size and surface area [14].

Elderly, children, pregnant women, those with chronic diseases, and those with low immunity represent the most vulnerable group due to the presence and variation of air pollution [10, 15–17]. Exposure to air pollution is a cause of cardiovascular disease, with a high association of particulate matter [9]. Cardiovascular diseases are a major public health topic, accounting for nearly 30% of worldwide mortality [18].

Research involving emerging countries is still very incipient [19, 20]. Although environmental pollution is a serious environmental problem in developing countries, few studies investigate the effects of PM_2.5‒10_ particulate matter due to PM_2.5_ monitoring constraints [13]. Given the high levels of PM_2.5_ pollution in Asia, Africa, and the Middle East, air pollution in Latin America receives little attention. However, between 1990 and 2013, annual averages of PM_2.5_ showed a high upward trend in parts of South America [21].

## 3. Materials and Methods

This is an ecological time-series study of hospital admissions for respiratory and cardiovascular diseases among children (5 years of age and under) and elderly (60 years of age and older) in the city of Manaus, State of Amazonas, Brazil, from 2008 to 2012. It has an area of 11,474 km^2^ (0.73% of the State area) with a total population of 1.8 million people and a demographic density of 158.06 inhabitants per square kilometres. The climate of Manaus is considered humid tropical with an annual average temperature of 27°C and relatively high air humidity. The seasons are relatively well defined regarding rain: winter is very rainy and summer is relatively dry. Due to the proximity of the Equator line, the heat is constant and there are no cold winter days [22]. The capital of the state of Amazonas, Manaus, was chosen because it represents the urban centre of the Amazon, marked by an intense process of urban expansion and population growth since the 1970s and a total accumulated deforestation until 2012 of 1,256.6 km^2^, equivalent to 10.9% of the city area.

Data on hospital admissions for respiratory diseases of residents of Manaus were collected from the Hospital Information System (HIS) for the analysed period, provided by the Department of Informatics of the Unified Health System (DATASUS). All hospitalizations for respiratory diseases (ICD-10th revision: J00 to J99) in children (0 to 5 years) and elderly people (60 years or older) and for cardiovascular diseases (ICD-10th revision: I00 to I99) in the elderly (60 years or more) in this period were selected for this study. Particulate matter concentrations (PM_2.5_ in *μ*g/m³), minimum temperature, and relative humidity of the air were used as exposure variables. These values were obtained through the Environmental Information System Integrated with the Environmental Health (SISAM).

Descriptive analyses of all variables of the study were carried out by calculating the values of central tendency and dispersion. To estimate the effects of the daily variation in the concentration of pollutants on the outcomes of interest, Poisson generalized linear models (GLM) were used to assess atmospheric pollution and hospitalization for cardiovascular and respiratory diseases in the city of Manaus.

The independent variables were the average daily levels of each pollutant, the minimum temperature (in °C), and the average relative humidity (in %). To address the effect of long-term seasonality, the number of sequential days elapsed (from 1 to 1827) was used in the analysis, and in the case of short-term seasonality, the days of the week (from 1 to 7, considering Sunday as the first day) were considered. The cubic spline natural straightener was used for smoothing long-term seasonality. The number of inflection points or the degrees of freedom of the straightener were chosen to minimize the sum of residual autocorrelation using the Akaike criteria [23]. Weekdays were regarded as categorical variables, with Sunday as baseline. Temperature and humidity entered the model as continuous variables.

For data with normal distribution, suppressing the covariates, a distributed lag model can take the following form [24, 25]:

(1) $$\begin{array}{c} \text{log}\left[E\left(Y_{t}\right)\right]=\alpha +\beta_{0}X_{t}+\cdots +\beta X_{t-q}+\varepsilon_{t}, \end{array}$$

where *X*_*t*-*q*_ is the environmental exposure of interest, for example, and air pollutants, *q* are the days before the estimated event, for example, hospital admissions. The total effect of environmental exposure is the sum of the estimated effects of subsequent *q* days. Therefore, it can be written as *β*_0_+⋯+*β*_*q*_. However, the high correlation between consecutive days makes individual estimates unstable for each *β*_*q*_.

To solve this problem of the days of the lag period analysed, a constraint factor has been imposed that causes the *β*_*q*_ to vary smoothly as a polynomial function. This polynomial distribution lag model has *q* days and *d* degrees of freedom (degree of polynomial) and may be restrictive depending on the degree of the polynomial used. As the central point of interest of this study is to estimate the effects of air pollutants on hospitalizations for cardiovascular and respiratory diseases and to explore the lag structure between exposure to these factors and the analysed outcome, polynomial distribution lag models were used because environmental exposures may cause deleterious effects in the case of hospitalizations for cardiovascular and respiratory diseases, on the day of exposure, one day after, or up to several days after exposure. Thus, hospitalizations that occur on a given day may be a result of exposure that occurred on the same day but also on other preceding days. To solve this problem, the analyses included a constraint factor that causes the coefficients to vary smoothly as a polynomial function of the days of the analysed lag period. The effects of exposure to pollutants and climate variables on a seven-day lag structure were estimated using third-degree polynomials that allow more stable estimates than those generated by models without the constraint factor. The effects of pollutant exposures on a 7-day lag structure (exposure day and up to 6 days after exposure) were estimated using third-degree polynomials that allow flexible and more stable estimates than unrestricted models [25, 26].

The results were presented as the increase in the number of hospitalizations and their respective 95% confidence intervals, for each day of the lag period analysed, due to increases of one interquartile in the concentration of pollutants. Interquartile variation can be defined as the difference between the third and first quartiles of the values of a given variable. The estimated percentage increase and its 95% confidence interval can be represented by

(2) $$\begin{array}{c} \text{increase}\left(\%\right)=\left(e^{\left(\beta_{q}*\text{IQV}\right)}-1\right)*100, \end{array}$$

where IQV is the interquartile variation and

(3) $$\begin{array}{c} \text{CI }95\%=\left(e^{\left(\left(\beta_{q}*\text{IQV}\right)\pm \left(1,96*{\text{SE}}_{q}*\text{IQV}\right)\right)}-1\right)*100, \end{array}$$

where SE*q* is the standard error for each *β*_*q*_.

Databases were prepared using the Statistical Package for the Social Sciences (SPSS) for Windows, version 21.0 (SPSS Inc., Chicago, United States). Regression analyses were performed with the S-PLUS 4.5 software.

## 4. Results and Discussion

During the analysed period, 501,493 hospital admissions occurred in the city of Manaus. Of this total, 50,278 were because of respiratory diseases and 34,582 due to cardiovascular diseases, representing 10.1% and 6.9% of the total number of hospitalizations, respectively. Among the hospitalizations for respiratory diseases, 29,582 (58.9%) were of children and 6,274 (12.5%) of elderly patients. Among the hospitalizations for cardiovascular disease, 17,778 (51.4%) occurred in elderly people.

Table 1 presents the descriptive parameters on hospitalization, level of PM_2.5_, minimum temperature, and average humidity. It is observed that for PM_2.5_, both the 25% percentile and the 50% percentile have the same value, showing inaccuracies in the measurement of this pollutant, as well as in the measurement of minimum temperature, which presented a minimum value of 1.5°C, which is impossible in a hot region such as the Amazon.

Table 2 presents the Spearman correlation matrix between PM_2.5_, temperature, humidity and hospitalizations for respiratory diseases in under-five children and cardiovascular and respiratory diseases in patients over 60 years of age.

The bivariate exploratory analysis of the correlations performed through the correlation matrix (Table 2) showed that no significant statistical correlations were observed between respiratory and cardiovascular diseases in patients over 60 years of age and respiratory diseases in under-five children and PM_2.5_, temperature, and air relative humidity.

**Figure 1:**
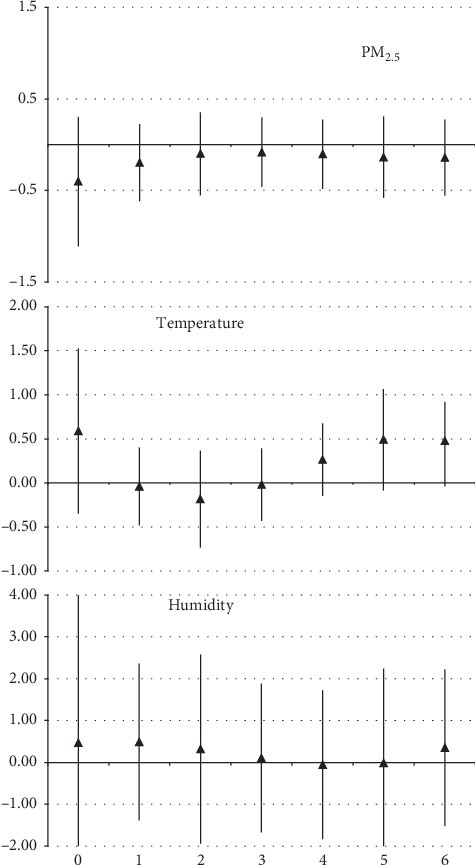
One interquartile range increase of daily mean level of PM_2.5_, using polynomial distribution, for hospitalizations due to respiratory diseases in children under 5 years in Manaus (AM), from 2008 to 2012.

In the statistical modeling with the GLM model, the estimated effects were not statistically significant in the case of respiratory diseases in under-five children. One interquartile range increase of daily mean level represented an increase in hospitalizations of −0.40% (95% CI: −1.11, 0.30), 0.59% (95% CI: −0.35, 1.52), and 0.47% (95% CI: −3.28, 4.21) for PM_2.5_, temperature, and humidity, respectively, as observed in Figure 1.

**Figure 2:**
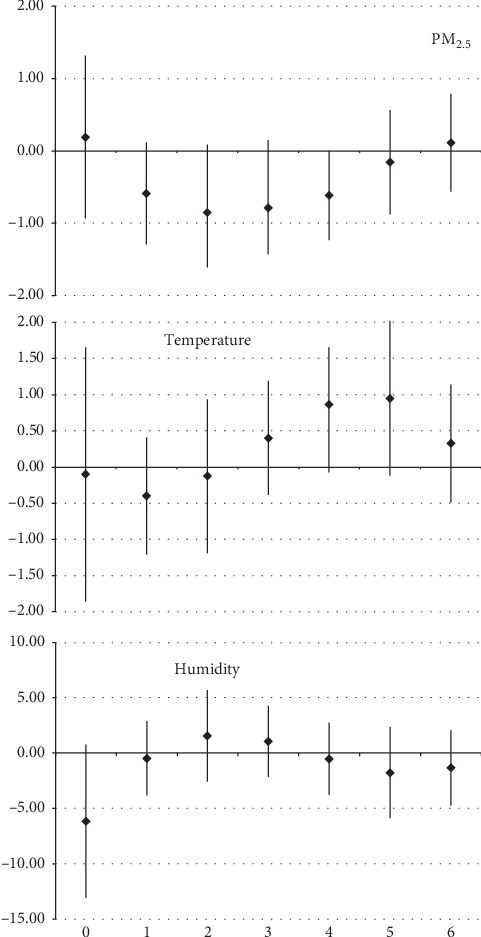
One interquartile range increase of daily mean level of PM_2.5_, for hospitalizations due to respiratory diseases in patients over 60 years of age in Manaus (AM), from 2008 to 2012.

In the case of respiratory diseases in patients with 60 years of age and over, the estimated effects were not statistically significant in the model. One interquartile range increase of daily mean level represented an increase of hospitalizations of 0.19% (95% CI: −0.93, 1.31), −0.10% (95% CI: −1.85, 1.65), and -6.17% (95% CI: −13.08, 0.74) for PM_2.5_, temperature, and humidity, respectively, as shown in Figure 2.

**Figure 3:**
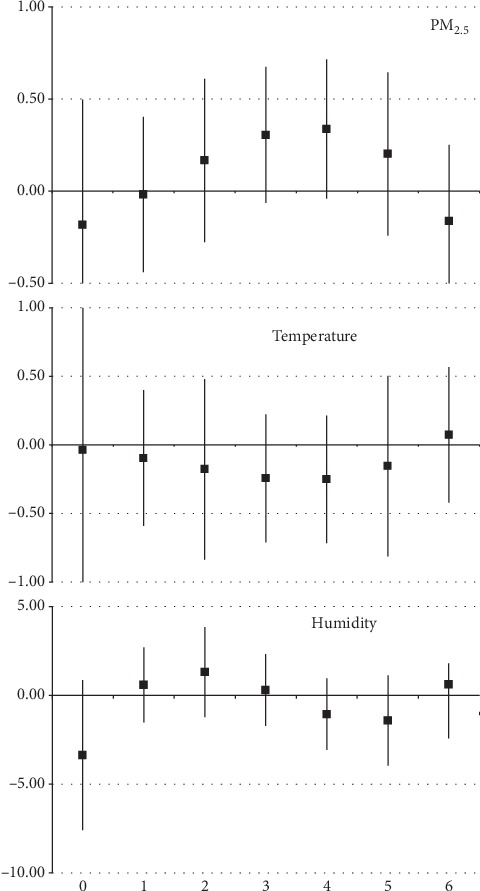
One interquartile range increase of daily mean level of PM_2.5_, for hospitalizations due to cardiovascular diseases in patients over 60 years of age in Manaus (AM), from 2008 to 2012.

For cardiovascular diseases and elderly, the estimated effects were not statistically significant. One interquartile range increase of daily mean level represented an increase of hospitalizations of −0.18% (95% CI: −0.86, 0.50), −0.04% (95% CI: −1.10, 1.03), and −3.37% (95% CI: −7.59, 0.85) for PM_2.5_, temperature, and humidity, respectively, as observed in Figure 3.

The time series did not find a significant association between PM_2.5_ concentrations, minimum temperature, or relative humidity and hospitalizations for respiratory diseases in under-five children and respiratory and cardiovascular diseases in patients over 60 years of age.

The first possible explanation for the inconsistencies found in the results is the way PM_2.5_ levels are measured. The satellite measurements provided by SISAM showed the same value (10 *μ*g/m^3^) for 70% of the studied days. It was also observed that that for PM_2.5_, both the 25% percentile and the 50% percentile had the same value, thus indicating inaccuracies in the measurement of this pollutant. The same happened with temperature, which presented a minimum value of 1.5°C, which is impossible in a hot region such as the Amazon. These possible measurement errors represent shortcomings in the time series that prevented the statistical model to find correlations between exposure to pollution and the diseases analysed in this study.

The results in the present study differ from other studies that showed the relationship between PM_2.5_ and the increased risk of hospitalization for cardiovascular disease in people over 60 years. The adverse effects of PM on hospitalizations for cardiovascular disease were correlated with the same day of pollutant increase (lag 0) and decreased the risk on subsequent days [27–30].

The present study also differs from the academic literature that shows an association between PM_2.5_ and the increase in hospitalization for cardiorespiratory diseases [31–33]. The literature shows that for children, an increase in the PM_2.5_ level increased the number of hospitalizations for respiratory diseases [34–37].

The academic literature also shows that the modification of the effects of PM_2.5_ by high temperatures on human health may be related to the direct or indirect response of the body to heat stress [38]. Body thermoregulation is directly linked to the circulatory regulation of an individual. Hot days can overload the body's temperature regulation system and alter the physiological response to toxic agents, increasing individual vulnerability [30].

We could not find any scientific articles that could support our findings. In fact, the lack of air quality monitoring seems to be a major element preventing a broader analysis of environmental and health conditions. However, as there is no air quality monitoring network in Manaus, we used the data provided by SISAM that comes from remote sensing (satellite). Those who research the effects of air pollution on health know that well-conducted studies and air quality monitoring programs require not only adequate methodology but also quality data on local pollution, weather conditions, and health information [39].

There are evidences that hospital admissions of children due to respiratory diseases in Manaus are more related to meteorological conditions and humidity than to exposure to smoke from the fires and to PM_2.5_ concentrations in the region. It is possible that the environmental characteristics inherent to the rainy season in the region play an important role in increased hospitalization rates of children due to respiratory diseases. There is a predominance of natural biogenic particulate emissions during the rainy season caused by the intense activity of biological organisms in the forest, which include fragments of plants and insects, pollen grains, fungi, algae, and fungal spores [40]. The presence of a ground monitoring system could provide more reliable data to confirm such hypothesis.

In any case, air pollution is pointed out by its systemic importance, in urban environments, because it has a synergic effect when allied to other exposure factors, potentiating the risks for respiratory or cardiovascular diseases. About the modernization process of urbanization, we must consider the relevance of the growth of the fleet of vehicles as emitters of atmospheric pollutants, which add to emissions of the operating thermoelectric plants and to the fires in the peripheral areas of Manaus. Thus, we highlight the possibility that the exposure of the population of Manaus to air pollutants has increased. From 2007 to 2011, the fleet of cars in the city increased by 65%. This is the highest rate among the ten most populous capitals. It is impossible, however, to quantify the seriousness of the situation due to the nonexistence of an atmospheric quality monitoring network in the city, different from other regions of Brazil.

About the hospitalization data, we only used the hospital admission authorizations (HAA) registered in DATASUS and comprised the information obtained in the hospitals linked to the Brazilian Unified Health System (SUS). It is worth noting that the HIS uses the HAA, and not the sick individual, as the unit of analysis. As the same individual can be hospitalized more than once or even not be hospitalized despite being ill due to limitations in the hospital structure, the use of hospitalizations as a proxy of the number of cases of illness is considered as fragility. However, HAA has been pointed out as one of the best information systems for health research [41]. We used data on hospitalizations in the SUS considering that hospitalization is the final stage of several visits to the emergency room, and several inhalations, because people only seek the emergency service and are hospitalized when their lung function is altered. On the other hand, the participation of the supplementary health system in the Amazon is not relevant in the region. There are cities where SUS is responsible for 100% of outpatient and hospital care, which indicates that the data used in this study have good population coverage.

Regarding the age groups selected for the study, several authors sustain that the most sensitive to exposure to air pollutants are under-five children and the elderly, as well as people with respiratory or cardiac diseases [42, 43]. However, other risk factors linked to sociological-sanitary conditions are also associated with the development of childhood respiratory diseases, especially in the first year of life [44, 45]. Of the two sensitive groups included in the present study, most of the groups with the highest prevalence of hospitalizations due to respiratory diseases occurred in the municipalities belonging to the “Arch of Deforestation,” composed of municipalities that extend from the state of Acre to the south of Maranhão and are characterized by extensive deforested areas and large concentrations of forest fires [46].

When time-series studies are used to assess the effects of pollution on health, they consider only the short-term impacts. A longer time series with more consistent information would enable the detection of small short-term effects on public health caused by exposure to air pollution [47]. Considering that the city of Manaus may be chronically polluted, the present results may underestimate the real damages.

As it is well known, epidemiological studies only provide indications of the associations between pollutants and human health. Based on the premises of this study, we hope that researchers are prompted to examine this subject plan research that can provide more conclusive results.

## 5. Conclusions

The present study found no significant association between PM_2.5_ concentrations, minimum temperature, relative humidity, and hospitalizations for respiratory diseases in children under 5 years and respiratory and cardiovascular diseases in those over 60 years.

Studies of the impact of air pollution on health using time series can contribute to obtain short-term effects of air pollution on respiratory and cardiovascular morbidity, supporting the control and warn actions to health services when pollution peaks occur. The realization of such studies depends on the continuous collection and availability of quality data. In this sense, efforts must be made to ensure the continuous collection of data and expansion of the air quality monitoring network, in the northern region of the country.

It should be emphasized that the measurement of climatic variables is also essential in all seasons to properly fit models and improve the results. On the other hand, efforts must also be undertaken by the health sector, in the acquisition and systematization of all information on hospital morbidity. Considering the seriousness of the situation of air pollution in the city of Manaus, the implementation of a computerized registry of outpatient clinics, especially children and older people, and improving the quality of data on air pollution are recommended to create possibilities for other research routes.

Remote sensing techniques are used as a complementary tool to surface measurements because they are indirect measurements, which explain the importance of long-term terrestrial-monitoring stations for integrated studies in the region.

We stress that it is a great challenge to describe conditions and trends in terms of environmental situations and exposures to which the population of Manaus is subjected. The results of this research can be used to evaluate the improvement of the quality of hospitalization and air pollution data. On the one hand, we must deal with the poor production of environmental indicators, and on the other hand, we must research the most different sources and documents for the description of these aspects in a city in which the municipal level follows the logic of production of information within the peculiarities of the municipality, especially for relatively new themes.

## Figures and Tables

**Table 1:** Basic descriptive parameters in Manaus, Amazonas, Brazil, from 2008 to 2012.

	Respiratory diseases		Cardiovascular diseases	PM_2.5_	Temperature	Humidity
	Children	Elderly	Elderly			
Mean	13.3	3.2	9.0	13.5	22.7	80.0
Standard deviation	8.1	2.1	4.0	9.1	4.0	15.1
Minimum	0.0	0.0	0.0	10.0	1.5	18.6
Maximum	50.0	13.0	30.0	117.4	37.3	99.2
25^th^	11.0	3.0	9.0	10.0	23.1	67.8
50^th^	11.0	3.0	9.0	10.0	23.8	85.7
75^th^	17.0	4.0	12.0	13.7	24.5	92.3

PM_2.5_: particulate matter (PM_2.5_ in *μ*g/m^3^).

**Table 2:** Spearman correlation matrix for the variables in the study.

	PM_2.5_	Minimum temperature	Humidity	RD ≤ 5 years	RD ≥ 60 years	CD ≥ 60 years
PM_2.5_	1.000					
Minimum temperature	−0.082	1.000				
Humidity	−0.550	−0.116	1.000			
RD ≤ 5 years	0.060	−0.086	0.003	1.000		
RD ≥ 60 years	0.006	−0.025	−0.007	0.148	1.000	
CD ≥ 60 years	0.004	0.012	0.030	0.159	0.237	1.000

PM_2.5_: particulate matter (*μ*g/m³); RD: respiratory diseases; CD: cardiovascular diseases.

## Data Availability

All the data reported in this manuscript have been deposited at Mendeley Data. Copies of this data can be obtained free of charge from https://data.mendeley.com/.
